# Commentary: Itsy Bitsy Spider…: Infants React with Increased Arousal to Spiders and Snakes

**DOI:** 10.3389/fpsyg.2018.00393

**Published:** 2018-04-05

**Authors:** Wolfgang Denzer

**Affiliations:** Wolfden Scientific Consultancy, Archena, Spain

**Keywords:** ancient threats, pupil dilation, infant, snakes, spiders

In a recent issue of this journal Hoehl et al. (2017) presented a paper dealing with infants' response to “ancestral threats” such as spiders and snakes. Their data, hypothesis and conclusion are disputable and discussed in the following.

The authors' hypothesis reads as follows: “Assuming an evolved preparedness to develop fear for ancestral threats, we predict increased pupillary dilation for spiders and snakes when compared to visually matched control stimuli that do not represent an ancestral threat to humans (i.e., flowers and fish).” Their experimental data are interpreted as supportive and consequently the following conclusion is presented: “In summary, our results support the notion of an evolved mechanism that is sensitive to spiders and snakes.” However, corroborating a hypothesis does not prove that the hypothesis was correct in the first place. Hoehl et al. combine two fundamental claims, namely that spiders and snakes posed a threat to primates and early humans, and secondly that pupil dilation is correlated with fear or negative stimuli.

Firstly, they do not provide any evidence from the zoological or paleontological literature how or why spiders would have been dangerous to primates. Nor do they discuss any publication that deals with the interaction of early humans or primates and snakes. While in the case of snakes information can be found (see for example Headland and Greene, 2011) such studies involving spiders are scarce or non-existent. Spiders do not pose a threat or medical risk for humans nowadays (Vetter and Isbister, 2008) and most probably never did during primate evolution and in particular during early human evolution. An alternative thesis regarding aversion to spiders based on cultural rather than evolutionary origins as proposed by Davey (1994) is not discussed.

Secondly, their interpretation of pupil dilation measurements to support their hypothesis is prone to criticism. In both of their experiments in Study 1 (within-participant study) the items are color-matched but they are not matched for shape. Fish are presented in a lateral view and flowers mainly in a frontal view. In contrast, the pictures of spiders are more complex than those of flowers and fish. The same is true for at least two of the snake pictures, both of which have their head pointing toward the observer (striking position). Already Hess and Polt (1964) and Kahneman (1973) have shown that pupil dilation correlates with the complexity of the task. As such the hypothesis presented by Hoehl et al. is not the only possible hypothesis. Another research group could employ the same experimental parameters in order to conclude that in infants increasing complexity of color-matched pictures triggers pupil dilation or that animals have a higher arousal potential than plants. Hence, the conclusion of Hoehl et al. does not hold as it is ambiguous and results can be interpreted in different ways.

While it appears that the within-participants Study 1 involving flowers and spiders supports their notion this is not true for the snake / fish experiment. The authors speculate that a carry-over effect was responsible for failing to produce the expected results and proceed with an additional between-participant study involving the same snake and fish pictures (Study 2) to verify their hypothesis. However, their interpretation of the results is inconsistent. In Figure 4 (Study 1) all values for pupil dilation stay below 0.2 mm on average. In Figure 5 (Study 2) all pupil dilation values within analysis time are above 0.2 mm for snakes while those for fish are mainly below 0.2 mm. This contradicts their assumption and conclusion that the infants “may have been aroused specifically by the snake stimuli but this response carried over to the visually closely matched fish.” If that had been the case, a more dilated pupil would have been expected to carry over and all values in study 1 should be above 0.2 mm. Their data rather indicate that the fish had a calming effect if a carry-over effect existed. Unfortunately, the corresponding between-participant study involving pictures of spiders and flowers was not conducted.

Finally it should be noted that studies by DeLoache and LoBue (2009) and LoBue et al. (2013) support the idea that children have a general affinity toward animals irrespective of whether they pose a threat or not. Children (9 months old) tried to grasp moving snakes from a screen, and even when presented with a live spider and snake (both caged) children (18–36 months old) did not express fear, but instead “demonstrated an avid interest.” The data presented by Hoehl et al. only partially support their hypothesis and certainly do not provide proof that the observed results can be related to an “***evolved*** [my emphasis] preparedness for developing fear of these ancestral threats.”

## Author contributions

The author confirms being the sole contributor of this work and approved it for publication.

## Conflict of interest statement

The author declares that the research was conducted in the absence of any commercial or financial relationships that could be construed as a potential conflict of interest.
